# Synbiotics suppress colitis-induced tumorigenesis in a colon-specific cancer mouse model

**DOI:** 10.1371/journal.pone.0216393

**Published:** 2019-06-26

**Authors:** Yasufumi Saito, Takao Hinoi, Tomohiro Adachi, Masashi Miguchi, Hiroaki Niitsu, Masatoshi Kochi, Haruki Sada, Yusuke Sotomaru, Naoya Sakamoto, Kazuhiro Sentani, Naohide Oue, Wataru Yasui, Hirotaka Tashiro, Hideki Ohdan

**Affiliations:** 1 Department of Gastroenterological and Transplant Surgery, Division of Medicine, Biomedical Sciences Major, Graduate School of Biomedical & Health Sciences, Hiroshima University, Hiroshima, Japan; 2 Department of Clinical and Molecular Genetics, Hiroshima University Hospital, Hiroshima, Japan; 3 Department of Surgery, Division of Molecular Oncology, Institute for Clinical Research, National Hospital Organization Kure Medical Center and Chugoku Cancer Center, Hiroshima, Japan; 4 Vanderbilt University Medical Center, GI medicine, Nashville, Tennessee, United States of America; 5 Natural Science Center for Basic Research and Development, Hiroshima University, Hiroshima, Japan; 6 Department of Molecular Pathology, Hiroshima University Institute of Biomedical and Health Sciences, Hiroshima, Japan; Future University, EGYPT

## Abstract

Although synbiotics may be effective in maintaining remission of inflammatory bowel disease, their anticarcinogenic effects are still debated. To address this issue, we evaluated the effects of synbiotics, probiotics, and prebiotics on tumorigenesis using a *CDX2P-Cre; Apc*^+/flox^ mouse model harboring a colon-specific *Apc* knock out, which develops adenoma and adenocarcinoma of the colon. Dextran sodium sulfate (DSS)-administration promoted colonic tumor development in *CDX2P-Cre*; *Apc*^+/flox^ mice, and these tumors were associated with loss of *Apc* heterozygosity, as confirmed by observation of well-differentiated adenocarcinomas with β-catenin accumulation in tumor cell cytoplasm. Synbiotics-treatment suppressed dextran sodium sulfate-induced colitis in *CDX2P-Cre*; *Apc*^+/flox^ mice, thereby reducing mortality, and inhibited tumorigenesis accelerated by DSS-administration. Conversely, neither probiotics nor prebiotics had any effect on inflammation and tumorigenesis. *Lactobacillus casei* and *Bifidobacterium breve* were detected in the fecal microbiota of probiotics-treated mice. Synbiotics-treatment suppressed DSS-induced expression of *IL-6*, *STAT-3*, *COX-2*, and *TNF-α* gene transcripts in normal colonic epithelium, indicating the possibility of suppressing tumor development. Importantly, these genes may be potential therapeutic targets in inflammation-associated colon cancer.

## Introduction

Individuals with inflammatory bowel disease have a 10- to 40-fold increased risk of developing colorectal cancer compared with the general population. This indicates that colitis-associated cancer develops from chronically persistently inflamed mucosa, and progresses through dysplasia to adenocarcinoma. Therefore, efficacious anti-inflammatory treatment can reduce or retard the development of colorectal dysplasia and cancer in inflammatory bowel disease [1–4]. Nonetheless, the mechanisms that link these chronic inflammatory states to colorectal cancer development are largely unknown. Experimental evidence suggests that chronic inflammation creates a favorable environment for colitis-associated cancer initiation and for tumor growth promotion and progression [5,6]. Noxious compounds released during chronic colon inflammation are thought to damage DNA and/or alter cell proliferation or survival, thereby promoting oncogenesis [1,2]. New insights that suggest a direct relationship between the DNA damage response and chromosomal instability (CIN) have been provided by *in vivo* studies [7,8]. Immune cells, which often infiltrate tumors and preneoplastic lesions, produce a variety of cytokines and chemokines that propagate a localized inflammatory response, and also enhance premalignant cell growth and survival by activating signaling pathways, such as those involving IL-6/STAT3, TNF-α, PGE2/COX-2, NF-κB, or MAPKs [5,8–12].

The pathogenesis of inflammatory bowel disease is related to inappropriate and exaggerated mucosal immune responses to constituents of the intestinal flora [13,14]. Dextran sodium sulfate (DSS)-induced colitis is a well-established animal model of mucosal inflammation that has been used in the study of ulcerative colitis pathogenesis and in preclinical studies [6,11,15]. DSS is known to be directly cytotoxic to cells at multiple levels, resulting in induction of colonic epithelium breakdown [6,16–20]. Exposure to gut flora leads to a significant increase in the expression of several proinflammatory cytokines, chemokines, nitric oxide, and inducible nitric oxide synthase [21–24]. Two inflammation-associated cancer mouse models induced by DSS have been reported. One is the *Apc*^MIN/+^ mouse, which shows increased intestinal adenoma and adenocarcinoma increase on DSS-administration [25]. Another model involves administration of azoxymethane (AOM) as a carcinogen and DSS to mice [6].

Previously, we demonstrated that *CDX2P 9*. *5-NLS Cre; Apc*^*+/flox*^ *(CPC;Apc)* mice develop adenomas and carcinomas mainly in the distal colon and rectum, together with a small number of cecum and small intestine adenomas [26]. In human colorectal carcinoma with the CIN phenotype, there is a frequent loss of heterozygosity at loci on chromosomes 5q, 17p, and 18q [27], whereas in *CPC;Apc* mice carrying constitutional, heterozygous, inactivating mutations in the *Apc* gene, the wild-type *Apc* allele is inactivated by loss of heterozygosity, indicating that CIN contributes to tumor progression.

“Synbiotics” (“syn” -together and “bios” -life) are a combination of probiotic bacteria and a growth-promoting prebiotic ingredient that are purported to exhibit synergism [28]. Several studies have shown that synbiotics might be effective for maintaining remission of inflammatory bowel disease in patients, and a previous review of synbiotics indicated possible inhibitory mechanisms in colon carcinogenesis [28–34]. However, the anticarcinogenic effect of synbiotics is ambiguous and still under debate.

In Japan, the *Lactobacillus casei* strain Shirota and *Bifidobacterium breve* strain Yakult have been marketed since 1935, and are common lactic acid bacteria which are available commercially throughout the world. The probiotics and prebiotics used in this study were chosen because they were found in Japan, are widely used worldwide as a general supplement reported to have good effects, and are readily obtainable [35,36].

In this study, we created a new mouse model that promoted tumor development by eliciting colitis in *CPC;Apc* mice, which experience spontaneous colon cancer. Using this model, we evaluated the impact of synbiotics, probiotics, and prebiotics, and examined the mechanism of tumorigenesis.

## Materials and methods

### Ethics statement

This study was performed in strict accordance with the Guide for the Care and Use of Laboratory Animals and the local committee for animal experiments. All animal protocols were approved by the Committee on the Ethics of Animal Experiments of Hiroshima University (Permit Number: 10–008). We checked the body weights of the mice every day, and euthanized them immediately after weight loss was detected. Surgery was performed under sodium pentobarbital anesthesia, and all efforts were made to minimize the suffering of the mice. Mice were euthanized by CO2 asphyxiation as per IACUC guidelines.

### Bacterial cells: Probiotics and prebiotics

In this study, the *Lactobacillus casei* strain Shirota and *Bifidobacterium breve* strain Yakult, were obtained from the Japan Collection of Microorganisms (Saitama, Japan), and were used as probiotics [35,36]. These strains were cultured in Gifu Anaerobic Medium broth (Nissui Pharmaceuticals, Tokyo, Japan) under anaerobic conditions using AnaeroPack (Mitsubishi Gas Chemical, Tokyo, Japan) at 37°C for 16 h. The harvested bacterial cells were washed twice with phosphate-buffered saline (PBS) and resuspended in PBS at a concentration of 1 × 10^8^ colony-forming units/mL. Suspensions were stored at -80°C until use. 4^G^-β-Galactosyl-sucrose (3.75 g/body; Ensuiko Sugar Refining. Co. Ltd, Japan) was used as a prebiotic [37].

### Animal model

Male *CPC;Apc* mice were used in this study in order to avoid sex bias.

To obtain *CPC;Apc* mice, 8-week-old *Apc*^*flox/flox*^ females were bred with male CDX2P 9.5-NLS Cre males. All mice were housed under specific pathogen-free conditions. Teklad Mouse Breeder Diet 8626 (Harland-Teklad) and automatically supplied water were provided to all mice used in tumorigenesis experiments. The breeding room was maintained at a constant temperature of 23°C±2°C, relative humidity of 50%±5%, 15–20 air changes per hour, and a 12-h light/dark cycle, with lights on at 8:00 am. Four or five mice were housed per cage with chopped wood bedding [38].

To confirm the mouse genotype, loss of *Apc* heterozygosity was assessed by multiplex PCR using the following primers: *Apc*-P3, 5ʹ-GTTCTGTATCATGGAAAGATAGGTGGTC-3ʹ; *Apc*-P4, 5ʹ-CACTCAAAACGCTTTTGAGGGTTGATTC-3ʹ; and *Apc*-P5, 5ʹ-GAGTACGGGGTCTCTGTCTCAGTGAA-3ʹ. The target (580S), deletion (580D), and wild-type alleles yielded products of 314 (P3 and P4), 258 (P3 and P5), and 226 bp (P3 and P4), respectively. The presence of the *CDX2* promoter region was assessed by PCR as previously described [26].

### Induction of chronic colitis in mice; synbiotic, probiotic, and prebiotic treatments; and general assessment of colitis and tumorigenesis

Acute colitis was induced in 7- to 8-week-old mice by administering filter-purified drinking water (Millipore Corp., Billerica, MA, USA) containing 1% (w/v) DSS (MW 36,000–50,000; MP Biomedicals, Solon, OH, USA) for 7 days. From day 7 onwards, the animals received normal drinking water. To induce chronic colitis, the mice were administered 1% DSS for 7 days during weeks 8, 11, 14, and 17 [6,15]. Synbiotics, probiotics, and prebiotics were orally consumed daily from 7 weeks to 20 weeks. Body weight, stool consistency, and fecal blood loss were recorded daily. The number of mice administrated drugs in this study was as follows; *CPC;Apc* mice (control group) was 8, treated with synbiotics was 9, administrated DSS was 8, administrated DSS and treated prebiotics was 7, administrated DSS and treated probiotics was 7, and administrated DSS and treated synbiotics was 8. At 20 weeks of age, the entire gastrointestinal tract of mice was removed immediately after euthanizing and flushed with ice-cold PBS. Intestinal tissue was sliced longitudinally, and the location, number, and diameters of polyps in the colon were recorded. The intestine was transferred to 10% buffered formalin to be processed for histopathological studies. Consistent with the histologic appearance, a hemispherical shape was assumed for large bowel polyps. We recorded the location, number, and diameter of large intestinal polyps.

### Disease activity score assessment and histopathological scoring

Body weight loss, stool consistency, and the presence of gross blood determined by fecal observation were assessed daily for each mouse to generate a weekly disease activity index (DAI), as described previously [39]. Each parameter was scored as shown in S1 Table. These scores were summed to obtain a DAI ranging from 0 to 12.

To assess DSS-induced colitis, colons were fixed in formalin and stained with hematoxylin and eosin (H&E). Sections were coded for blind microscopic assessment of inflammation (DSS-induced colitis). Histologic scoring was performed based on three parameters, i.e., the severity of inflammation, crypt damage, and ulceration, as described previously [39], with scores shown in S2 Table. The values were summed to give a histological score (maximum 11). At minimum, two sections of different parts of the distal colon per animal were scored.

### Immunohistochemistry

We performed immunohistochemical analysis as described previously [40]. Anti-β-catenin (BD Transduction Laboratories), rabbit monoclonal anti-CDX2 (clone EPR2764Y; Nichirei, Tokyo, Japan), rabbit polyclonal anti-p53 (NCL-p53-CM5; Leica Biosystems, Newcastle, UK), and rabbit monoclonal anti-Ki-67 (ab1667, Abcam plc, Cambridge, UK) antibodies were used at dilutions of 1:2,000, 1:1,000, 1:200, and 1:100 (final concentration, 5 μg/mL), respectively. The β-catenin, CDX2, p53, and Ki-67 staining positivity rates in the tumor area and normal colon epithelial cells were quantified using Image J. [41, 42]

### Total RNA extraction and quantitative real-time reverse transcription-PCR analysis

To assess the effect of DSS and synbiotics administration on gene transcription related to inflammation and carcinogenesis in background mouse mucosa, we performed quantitative RT-PCR using total RNA extracted from mouse colon epithelium. Total RNA was extracted from mouse normal colon epithelium using an RNeasy kit (Qiagen). Quantitative real-time PCR was performed as described previously [43].

We used commercially available *IL-6*, *STAT3*, *NF-κB*, *PGE-2*, *COX-2*, and *TNF-α* real-time RT PCR primers from Qiagen (product numbers: PPM03015A, PPM04643F, PPM26197A, PPM03647E, PPM30180A, and PPM03113G-200). The primer sequences used for amplification of *β-2m* (microglobulin) as an internal control were as follows: sense 5ʹ-TGGTCTTTCTGGTGCTTGTC-3ʹ, anti-sense 5ʹ -GTATGTTCGGCTTCCCATTC-3ʹ.

### Fecal bacteriological examinations

Feces were obtained directly from the colons of six mice in each treatment group to investigate the effect of *L*. *casei* and *B*. *breve* strains on the gut microbiota. Fecal samples for bacteriological analysis were acquired from pre- and post-treated mice at 20 weeks of age. Immediately after defecation, fecal samples were weighed and suspended in nine volumes of RNAlater (Ambion Inc., Austin, TX, USA). The preparations were then incubated for 10 min at room temperature. For RNA stabilization, fecal homogenate (200 μL) was added to 1 mL of sterilized PBS and centrifuged at 5,000 × *g* for 10 min. The supernatant was discarded and the pellet stored at -80°C until RNA extraction. RNA was isolated using a modification of the acid guanidinium thiocyanate-phenol-chloroform extraction method. The resulting nucleic acid fraction was suspended in 1 mL of nuclease-free water (Ambion) [44,45]. Bacterial numbers were determined by reverse transcription-quantitative polymerase chain reaction (RT-qPCR). A standard curve was generated from RT-qPCR data (using the threshold cycle [C_T_] method) and the corresponding cell count, which was determined microscopically with 4,6-diamidino-2-phenylindole (Vector Laboratories, Burlingame, CA) staining for the dilution series of the standard strains [46]. To measure the bacterial populations in each sample, three serial dilutions of extracted RNA were used for RT-qPCR. C_T_ values in the linear range of the assay were applied to the standard curve to obtain the corresponding bacterial cell count in each nucleic acid sample and then converted to the number of bacteria per sample. The specificity of the RT-qPCR assay using group- or species-specific primers was determined as described previously [44,45].

### Statistical analysis

All data are expressed as means ± standard deviations (SDs). Statistical significance was assessed using the Mann-Whitney U test, chi-square test, unpaired t test or Fisher’s exact test. Kruskal-Wallis analysis was used as a nonparametric test of multiplicity. The data were considered statistically significant at *P* **<** 0.05. All statistical analyses were performed using JMP 10 software (SAS Institute Inc., Cary, NC, USA).

## Results

### DSS-administration promotes colonic tumor development in a *CPC;Apc* mouse model and the tumors were caused by a loss of *Apc* heterozygosity

We investigated the effect of DSS-induced intestinal inflammation on large intestine tumorigenesis using *CPC;Apc* mice. We compared a DSS-administration group with a control group for the appearance of colon, cecum, and small intestine tumors. To assess loss of *Apc* heterozygosity, we performed *Apc* genotyping on the tumor, normal colon epithelium, and proximal small intestine.

**Fig 1:**
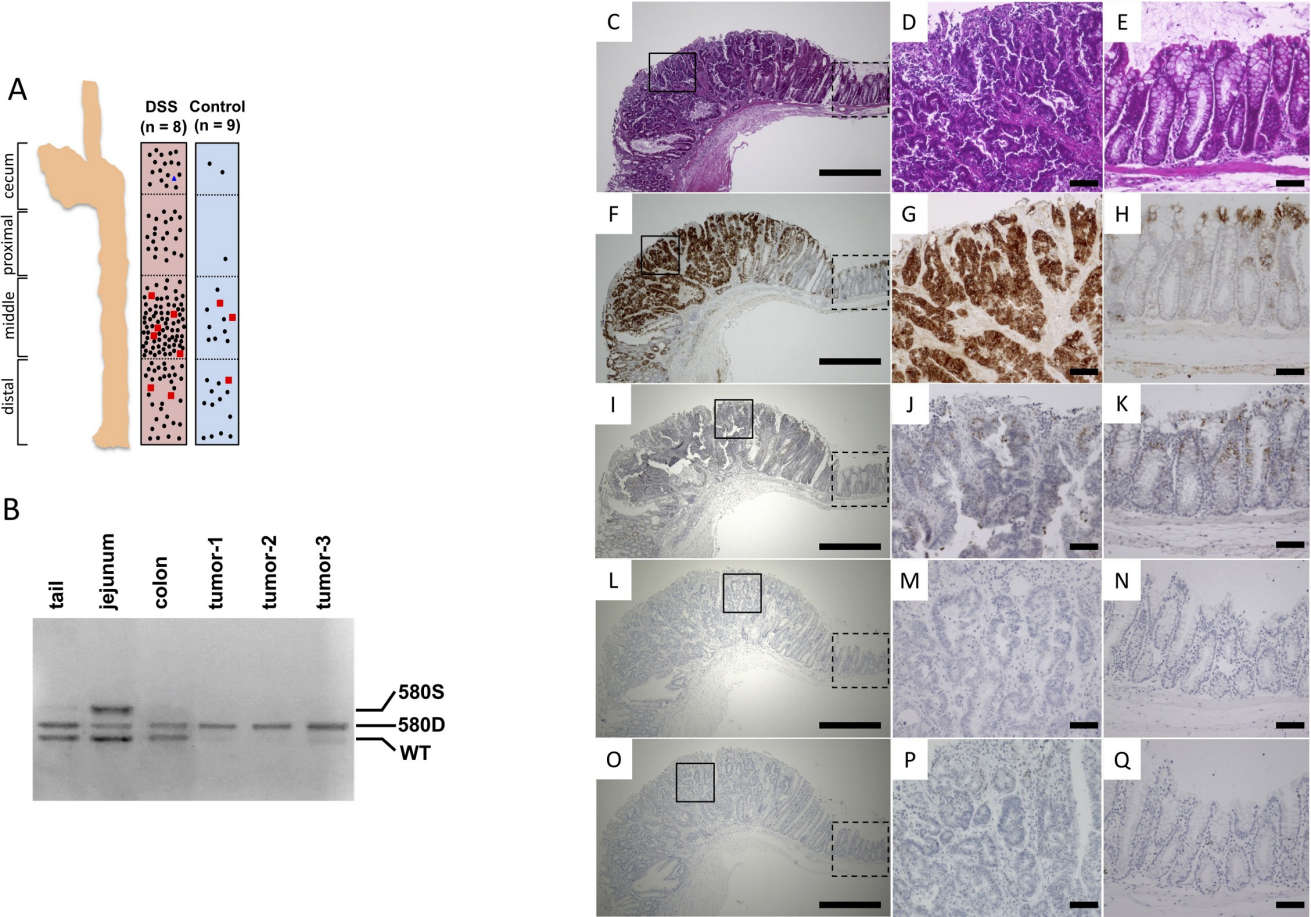
Evaluation of tumor formation and histological analysis. (A) Comparison of tumor number and site of occurrence in the large intestine between DSS-administered *CPC;Apc* mice and control mice. Solid circles indicate a tumor of 5 mm or more and less than 10 mm. Blue triangles indicate a tumor less than 5 mm. Red squares indicate a tumor of 10 mm or more. (B) Estimation of *Apc* loss of heterozygosity by multiplex PCR. Histological analysis of tumors in DSS-administered *CPC;Apc* mice. Hematoxylin and eosin-stained (C, D, E) and immunohistochemical staining of β-catenin (F, G, H), CDX2 (I, J, K), p53 (L, M, N), and Ki-67 (O, P, Q). (C, F, I, L, O: 40×, box with a solid line indicates a tumor; box with a broken line indicates normal colon epithelium. D, G, J, M, P: tumor 200×. E, H, K, N, Q: normal colon epithelium 200×.

Tumor number was increased in the DSS-administration group; however, there was no significant difference between the treatment and control groups with regards to maximum tumor diameter (tumor number [DSS vs. control]; 4 vs. 20; *P* = 0.002, tumor maximum diameter; 6 mm vs. 5.5 mm; *P* = 0.608) (Fig 1A). In the control group, tumors generally did not develop in the proximal large intestine; however, in DSS-administered mice, tumors developed in the proximal region at almost the same frequency as in the distal colon (Fig 1A). These tumors also showed a loss of *Apc* heterozygosity (Fig 1B).

### Tumor induction by DSS-administration was confirmed by the presence of well-differentiated adenocarcinomas with β-catenin accumulation in tumor cell cytoplasm

The tumors of DSS-administered mice had nuclear atypia and maintained the duct structure. Almost no infiltration into the submucosal layer was observed (Fig 1C–1E). a high accumulation of β-catenin was observed in the tumor cell cytoplasm, whereas normal colon epithelium in the mucosal crypt stained weakly for this marker (Fig 1F–1H). Immunostaining for CDX2 showed moderate staining in both tumor cells and normal colon epithelium cells (Fig 1I–1K), indicating well-differentiated tumors. Immunostaining for p53 produced light staining in both tumor and normal colon epithelium (Fig 1L–1N). Immunostaining for Ki-67 generally showed no staining in either tumor or normal colon epithelium (Fig 1O–1Q). On the basis of the histological findings, the tumors elicited by DSS-administration were well-differentiated adenocarcinomas with low invasive behavior and low growth potential at the time of sacrifice (20 weeks of age). The analysis of immunostaining positivity rates using ImageJ indicated that β-catenin, CDX2, p53, and Ki-67 were present in, respectively, 9.6%, 22%, 5%, and 3% of normal colon epithelial tissue. In contrast, they were present in, respectively, 88%, 30%, 10%, and 2% of tumor tissue.

### Synbiotics-treatment suppresses the symptoms of colitis induced by DSS, resulting in reduced mortality

**Fig 2:**
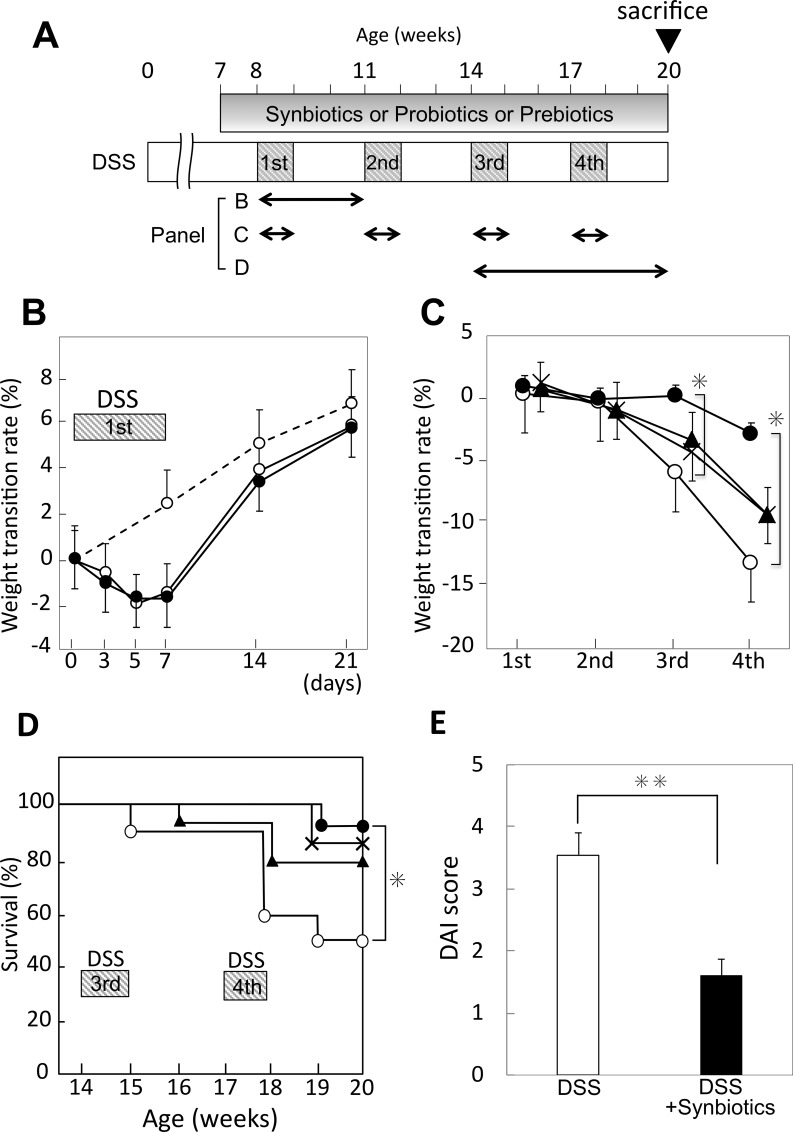
Administration schedule of DSS, probiotics and prebiotics. **Evaluation of body weight change and survival of mice and intestinal inflammation.** (A) Timetable of DSS-administration and drug-treatment with probiotics and prebiotics. (B) Weight transition for DSS-administration during course 1 (day 0–21, open circle and broken line: control, open circle and solid line: DSS-administered mice, solid circle and solid line: DSS-administered and synbiotics-treated mice). (C) Weight change during each DSS-administration course (1^st^ to 4^th^) in mice administered DSS and treated with probiotics and/or prebiotics (open circle: DSS-administration only, solid circle: DSS-administered and synbiotics-treated mice, cross: DSS-administration and probiotics-treatment, solid triangle: DSS-administration and prebiotics-treatment). (D) Percent survival of each group, with treatments indicated by the same symbols shown in (C).”(E) Disease activity index (DAI) of DSS-administered mice and mice administered DSS and treated with synbiotics. *: *P* < 0.01, **: *P* < 0.001.

To evaluate the severity of colitis, we measured changes in the body weight, survival rate, and colitis status of the mice using DAI scoring based on a combination of weight loss, rectal bleeding, and stool consistency. We evaluated the effect of one course of DSS-administration (Fig 2B), observing a weight loss of up to 2% in the DSS-administration group compared with the control. After discontinuation of DSS-administration, there was an immediate gain in weight. Therefore, we evaluated the change in body weight from day 0 to day 7, because day 7 represented the nadir of body weight. Over the course of administration, mice receiving DSS showed increased weight loss. Weight loss during the four courses of DSS-administration was 10% or more. In contrast, during the courses, synbiotics-treatment significantly suppressed weight loss by 5% or less (*P* < 0.05) (Fig 2C). In survival rate analysis, the DSS-administration group showed 50% mortality related to colitis or tumor. In contrast, a significantly lower mortality rate (10%) was observed in the DSS-administered mice receiving synbiotics-treatment (Fig 2D) (*P* = 0.04). On the other hand, probiotics and prebiotics alone resulted in a slight decrease in weight loss and a tendency to improve survival rate compared to treatment with DSS alone, but this difference was not significant. Synbiotics, administered to DSS-challenged mice, reduced DAI scores by 56% compared to those for animals that received DSS alone (Fig 2E) (DSS vs. DSS + synbiotics; 3.6 ± 0.35 vs. 1.6 ± 0.27, *P* < 0.001).

### Synbiotics-treatment inhibits tumor development accelerated by DSS-administration in a *CPC;Apc* mouse model

**Fig 3:**
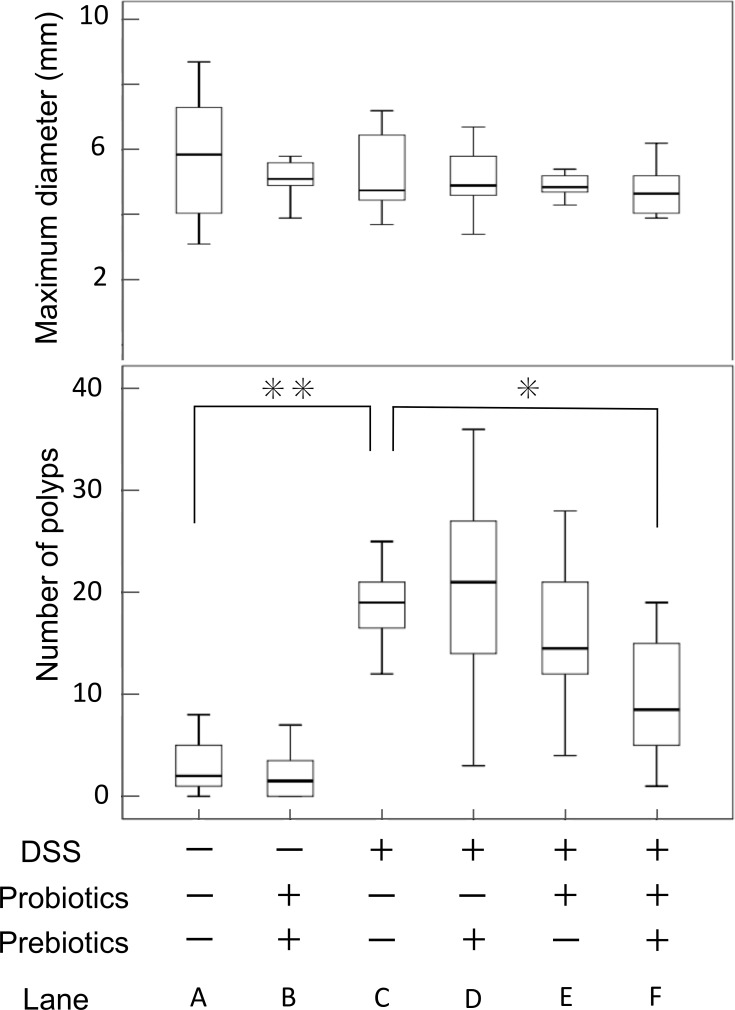
Comparison of tumor number and maximum tumor diameter. (A) *CPC;Apc* mice [average tumor number, average tumor maximum diameter (n = 8); 4.0, 5.9], (B) *CPC;Apc* mice + synbiotics [average tumor number, average tumor maximum diameter (n = 9); 3.5, 5.0], (C) *CPC;Apc* mice + DSS [average tumor number, average tumor maximum diameter (n = 8); 19.5, 4.4], (D) *CPC;Apc* mice + prebiotics (average tumor number, average tumor maximum diameter (n = 7); 21, 4.6), (E) *CPC;Apc* mice + probiotics [average tumor number, average tumor maximum diameter (n = 7); 14, 4.6], (F) *CPC;Apc* mice + synbiotics [average tumor number, average tumor maximum diameter (n = 8); 8.2, 4.5]. *: *P* = 0.01, **: *P* = 0.002.

We investigated tumorigenesis in *CPC;Apc* mice with or without DSS-administration, and in the DSS-administration + probiotics- and prebiotics-treatment groups. There was no significant difference between *CPC;Apc* mice in the synbiotics treatment and those in the non-treatment groups regarding tumor number (*P* = 0.379) and maximum tumor diameter (*P* = 0.509) (Fig 3).

No significant differences were observed in maximum tumor diameter among the experimental groups. However, there was a significant reduction (42%) in tumor number in the synbiotics-treatment group compared with the group administered DSS alone (DSS + synbiotics vs. DSS; 8.2 vs. 19.5: *P* = 0.01). There was no significant difference in tumor number in the probiotics-alone group or the prebiotics-alone group compared with the DSS-administration group (Fig 3).

### Synbiotics-treatment suppresses the inflammation of normal colon mucosa induced by DSS-administration

**Fig 4:**
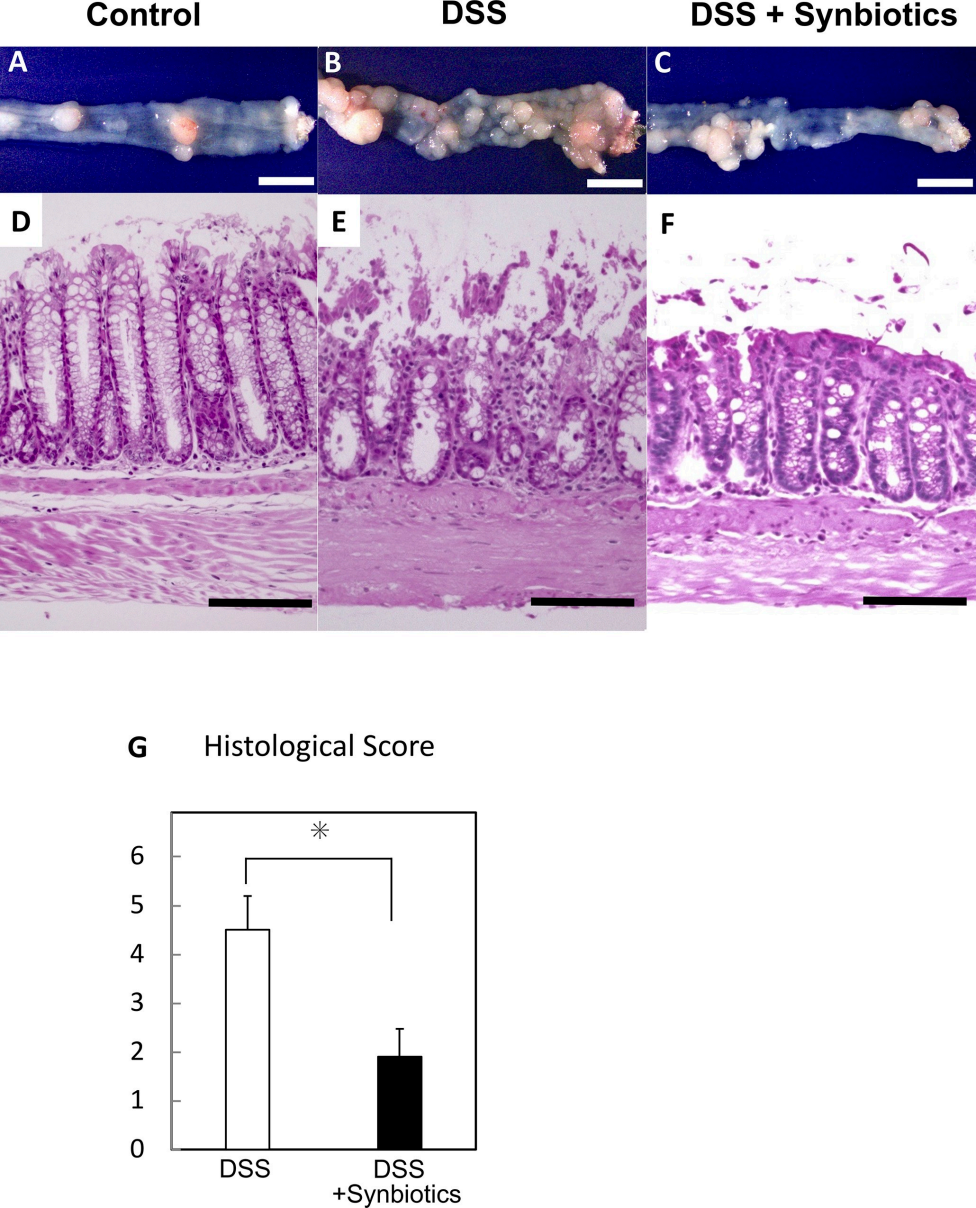
Analysis of background inflammation in the normal colon epithelium of DSS-administered and synbiotics-treated *CPC;Apc* mice using hematoxylin and eosin (H&E) staining and histological score. (A) control; *CPC;Apc* mouse. (B) *CPC;Apc* mouse administered DSS. (C) *CPC;Apc* mouse administered DSS with synbiotics-treatment (yellow scale 1 cm). H&E staining of normal colon epithelium (D; control, E; DSS-administered mouse, F; mouse administered with DSS and treated with synbiotics: ×200, black scale 100 μm) in *CPC;Apc* mouse. (G) Estimation of histological score of colon epithelium inflammation. (DSS vs. DSS + synbiotics; 4.5 ± 0.7 vs. 1.9 × 0.6, *P* < 0.01). *: *P* < 0.01.

Histological analysis of the large intestine indicates that tumor development was increased by DSS-administration and suppressed by simultaneous synbiotics-treatment (Fig 4A–4C). In addition to weight transition rate, survival rate, and DAI scoring, we estimated background mucosa inflammation histologically.

Although H&E staining of normal epithelium in the control group revealed no obvious inflammation of the background normal mucosa (Fig 4D), the DSS-administered group showed strong inflammation and mucosal damage, including strong inflammatory cell infiltration and an intermediate-to-high degree of erosion (Fig 4E). The DSS-administration + synbiotics-treatment group showed mucosal damage and moderate inflammatory cell infiltration and erosion (Fig 4F). To evaluate mouse colitis, we estimated the severity of colon inflammation, including crypt damage and ulceration, in the H&E-stained specimens. Synbiotics-treatment under DSS-administration decreased the inflammation score compared with DSS-administration alone (Fig 4G) (DSS + synbiotics vs. DSS; 1.9 ± 0.57 vs. 4.5 ± 0.69, *P* < 0.01).

### *Lactobacillus casei* and *Bifidobacterium breve* are present in the fecal microbiota of mice treated with synbiotics

The analysis of fecal microbiota shows that both *L*. *casei* and *B*. *breve* were present in the treatment group, but not in the non-treatment group (Table 1). Additionally, analysis of other anaerobic bacteria revealed no significant changes in the bacterial population (Table 1).

**Table 1:** Presence of *Lactobacillus casei* strain Shirota and *Bifidobacterium breve* strain Yakult and changes in the intestinal flora in mouse colon under administration of dextran sulfate sodium (DSS), synbiotics, *Lactobacillus* alone, and oligosaccharide alone.

Treatment group	a	b	c	d	e	f
	control	DSS(-)/syn	DSS(+)	DSS(+)/pro	DSS(+)/pre	DSS(+)/syn
mice number (n)	3	3	6	3	4	6
**Total bacteria**	9.7 ± 0.6	10.0 ± 0.4	9.1 ± 0.8	9.5 ± 0.4	10 ± 0.4	9.1 ± 0.6
**Obligatory anaerobe**						
*Clostridium coccoides* group	8.9 ± 1.3	9.6 ± 0.5	8.5 ± 0.8	8.9 ± 1.0	9.8 ± 0.6	8.5 ± 0.5
*C*. *leptum* subgroup	8.3 ± 1.1	8.7 ± 0.5	8.1 ± 0.5	9.1 ± 0.9	8.6 ± 0.5	8.3 ± 0.5
*Bacteroides fragilis* group	7.5 ± 0.4	8.1 ± 0.4	7.3 ± 1.0	7.8 ± 0.8	7.9 ± 0.3	7.7 ± 0.8
*Bifidobacterium*	7.9 ± 0.8	9.0 ± 0.1	8.0 ± 1.1	8.7 ± 1.2	8.4 ± 1.3	8.3 ± 1.0
*Atopobium* cluster	7.7 ± 0.5	9.0 ± 0.7	8.5 ± 0.9	8.0 ± 1.0	8.1 ± 0.3	8.4 ± 0.9
*Prevotella*	7.2 ± 0.5	8.0 ± 0.9	7.0 ± 0.6	7.5 ± 0.9	7.8 ± 0.6	7.6 ± 0.8
*C*. *perfringens*	<2.3	<2.3	<2.3	<2.3	4.3 ± 0	<2.3
**Facultative anaerobe**						
Total *Lactobacillus*	8.9 ± 0.5	8.9 ± 1.0	7.0 ± 1.1	7.9 ± 1.1	7.2 ± 0.3	7.4 ± 1.3
*L*. *gasseri* subgroup	8.4 ± 0.9	8.5 ± 1.5	6.4 ± 1.2	7.8 ± 1.2	6.6 ± 0.9	6.9 ± 1.5
*L*. *brevis*	3.4 ± 0.1	3.1 ± 0.5	<2.3	2.9 ± 0	<2.3	<2.3
*L*. *casei* subgroup	<3.0	7.0 ± 1.2	<3.0	5.8 ± 0.6	<2.9	5.4 ± 1.4
*L*. *fermentum*	<4.0	<4.0	<4.0	<4.0	<4.0	<4.0
*L*. *fructivorans*	<2.3	<2.3	<2.3	<2.3	<2.3	<2.3
*L*. *plantarum* subgroup	<2.4	2.8 ± 0.1	<2.4	<2.4	<2.4	<2.4
*L*. *reuteri* subgroup	8.3 ± 0.4	7.9 ± 0.6	6.6 ± 1.3	6.8 ± 1.3	5.8 ± 0.3	6.1 ± 1.2
*L*. *ruminis* subgroup	8.1 ± 0.6	8.0 ± 0.6	6.1 ± 0.8	7.0 ± 0.9	7.0 ± 0.3	6.4 ± 1.2
*L*. *sakei* subgroup	6.6 ± 0	5.5 ± 0.4	4.6 ± 1.0	5.0 ± 1.2	3.7 ± 0.4	4.4 ± 0.3
*Enterobacteriaceae*	5.3 ± 0	5.5 ± 0	5.0 ± 0.8	5.2 ± 0.4	4.8 ± 0	5.8 ± 0.3
*Enterococcus*	7.6 ± 0.7	7.4 ± 0.5	6.3 ± 0.4	6.4 ± 0.9	6.6 ± 0.2	6.6 ± 0.6
*Staphylococcus*	4.4 ± 0.3	4.7 ± 0.2	4.4 ± 0.1	4.5 ± 0.7	5.2 ± 0.4	5.0 ± 1.1
**Aerobes**						
*Pseudomonas*	<2.9	<2.9	<2.9	<2.9	<2.9	<2.9
*Lactobacillus casei* strain Shirota	<4.9	7.0 ± 1.2	<4.9	5.8 ± 0.6	<4.9	5.8 ± 0.6
*Bifidobacterium breve* strain Yakult	<5.0	7.3 ± 1.2	<5.0	6.0 ± 0.1	<5.0	6.2 ± 1.0

Mean bacterial counts (log10 cells/g) per 1 g of feces from 3–6 mice are indicated in each group.

Because the *L*. *casei* subgroup contains the *L*. *casei* strain Shirota, it was detected in the administration group. The *L*. *brevis*, *L*. *ruminis*, and *L*. *sakei* subgroups showed a decrease with DSS administration, although the differences were not significant.

### DSS-induced expression of IL-6, STAT-3, COX-2, and TNF-α gene transcripts in normal colonic epithelium was suppressed by synbiotics-treatment

**Fig 5:**
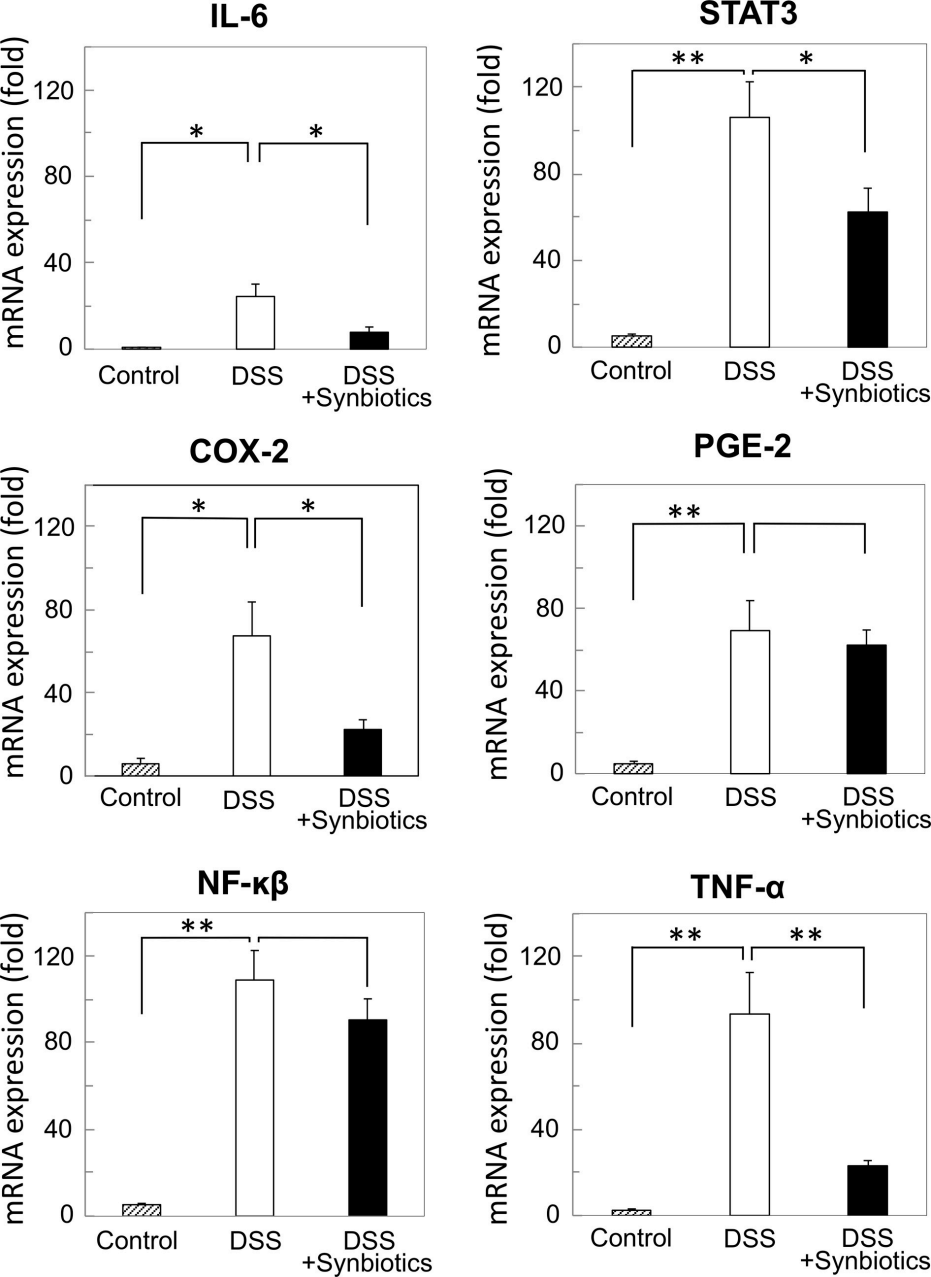
Expression analysis was performed for inflammation- and tumorigenesis-associated genes in normal colon epithelium by quantitative real-time PCR. Gene expression of total RNA samples from 20-week-old *CPC;Apc* mice (C: control, n = 8), 20-week-old DSS-administered *CPC;Apc* mice (D: DSS, n = 8), and 20-week-old *CPC;Apc* mice administered DSS and treated with synbiotics (DS: DSS + synbiotics, n = 8) was analyzed using commercial high-density oligonucleotide arrays. *: *P* < 0.05, **: *P* < 0.001.

Quantitative RT-PCR using total RNA extracted from mouse colon epithelium showed that, in the DSS-administration group, expression of IL-6, STAT3, COX-2, PGE-2, NF-κB was significantly increased by approximately 22- to 110-fold compared to that in the control by DSS administration. Synbiotics treatment significantly reversed the upregulation of IL-6 (63%), STAT3 (41%), COX-2 (66%), and TNF-α (73%) (Fig 5).

## Discussion

Colorectal cancer in mice is chemically induced with AOM, and the most-used model of colitis-associated colon cancer is induced with a combination of AOM and DSS [6]. To mimic known mechanisms underlying colitis and cancer in humans, genetically engineered mouse models have been created, of which *Apc*^MIN/+^ mice were among the first, although in this model tumor development was mostly limited to the small intestine [25]. Previously, we showed that intestine-specific *caudal*-related homeobox transcription factor *CDX2* elements confer colon epithelium-preferential transgene expression in the adult mouse, and that mice carrying a *CDX2P-NLS Cre* recombinase transgene and a floxed *Apc* allele developed colorectal adenomas and carcinomas [26]. Morphologic and molecular studies of the mouse tumors revealed their similarity to human colorectal tumors, suggesting that mice in which the *CDX2P*-*NLS Cre* transgene is used to target *Apc* (*CPC-Apc*), and other genes of interest such as *K-ras* and *Tgfbrt2*, simultaneously can be used for studies in colitis-induced colorectal cancer development. In this study, we created a new inflammation-associated colon cancer mouse model by treating *CPC;Apc* mice with DSS, characterized by *Apc* conditional knockout with a background of CIN. Our data demonstrated the inhibitory effects of synbiotics on tumor development through suppression of colitis using *CPC;Apc* mice. Tumor occurrence was elicited by DSS-promoted colitis, although tumor growth was not promoted. These observations are similar to the findings of a previous study using an *Apc*^MIN/+^ mouse model [25], in which background colitis was strongly involved in tumor development. Furthermore, as the *CPC;Apc* mouse model develops adenocarcinoma in a CIN background, these observations suggested that colon epithelium inflammation may promote tumor development through an effect on CIN.

Regarding the roles of synbiotics in colon cancer prevention, the current study demonstrated that synbiotics-treatment in *CPC;Apc* mice had no effect on tumorigenesis in terms of either tumor number or maximum diameter without intestinal inflammation induced by DSS. One possible explanation is that the mice were bred in a specific pathogen-free environment that maintained a constant balance of intestinal bacteria, resulting in a minimal effect of synbiotics in the mouse model of spontaneous carcinoma with colon-preferential *Apc* inactivation. In contrast, the human intestinal environment is exposed to various stresses, which cause aggravation of the intestinal environment and colitis [34]. Based on this background, we analyzed the impact of synbiotics on carcinogenesis induced by colitis. We demonstrated that treatment with synbiotics suppressed enteritis more effectively than administration of either *Lactobacillus* or oligosaccharides alone, thereby inhibiting inflammation-induced carcinogenesis in mice that reproduced an environment close to that of human colon carcinogenesis.

While previous studies have reported the effects of inflammation and intestinal bacteria on tumorigenesis [29,47], this inflammation-induced colon cancer mouse model based on CIN is considered a more useful model to investigate the carcinogenesis of colon for two reasons. First, this model does not require the use of chemicals such as carcinogens. When using carcinogens such as mutation inducers, the evaluation of genes associated with certain phenotypes might be difficult. The *CPC;Apc* mouse model is considered to offer a more precise analysis of tumor development because it involves just a single mutation (*Apc*). Second, the model enables observation of colon cancer development. Previous reports showed only small intestine adenoma or adenocarcinoma in mouse models of spontaneous intestinal cancer such as the *Apc*^MIN/+^ mouse, whereas the present model is considered to be superior in that it more closely reproduces the environment of human colon cancer.

We detected *Lactobacillus* in the feces of mice in the *Lactobacillus* treatment group, indicating that these bacteria reached the large intestine and persisted there. However, there was no significant change in other bacterial flora following synbiotics-treatment, suggesting that the administered *Lactobacillus* had a direct anti-inflammatory effect on the colonic mucosa. Previous studies have demonstrated that using probiotics and prebiotics in combination reduced the fecal pH of mice and increased the amounts of short-chain fatty acids, thereby preventing mucosal damage, including that of the colonic crypt cells, and further promoting regeneration [48,49]. Although we did not perform the relevant evaluations in the present study, it is believed that a combined administration of probiotics and prebiotics inhibits mucosal damage through the abovementioned mechanism. In addition, *L*. *brevis* and bacteria in the *L*. *ruminis* and *L*. *sakei* subgroups showed a decrease associated with mucosal disorder following DSS-administration, and this possibly affected the acceleration of tumorigenesis. Because the absence of *L*. *ruminis* has been reported to be correlated with lactate and butyrate contents in fecal waters [50], our observations can be considered compelling evidence of intestinal environmental change caused by DSS-administration. There was no significant change in the bacteria of the intestinal microbial flora in both the *Lactobacillus*-alone and oligosaccharide-alone groups, and thus other factors must be considered to explain the effect of oligosaccharide treatment on the intestinal mucosa.

Through quantification of the expression levels of gene transcripts associated with inflammation and tumorigenesis, we found that the expression of genes associated with inflammation, such as *IL-6*, *STAT3*, *NF-κB*, *PGE-2*, *COX-2*, and *TNF-α*, increased in the DSS-administration group. Among these genes, the expression of *IL-6*, *STAT3*, *COX-2*, and *TNF-α* was decreased in the synbiotics-treatment group with DSS administration. IL-6, STAT3, COX-2, and TNF-α have been reported to be associated with tumorigenesis [9,25,51–53], which was similarly demonstrated in the present analysis using *CPC;Apc* mice. Thus, tumor suppressive mechanisms that involve suppression of the transcripts of these genes are considered useful subjects for future therapeutic research. For example, antibody drugs for each of the gene products have already been developed; the anti-TNF-α antibody drug is infliximab, and IL-6 is targeted by the anti-IL-6 antibody tocilizumab as well as COX-2 inhibitors. These drugs may be expected to suppress tumor development. COX-2 inhibitors and NSAIDs have been shown to reduce the risk of death from colon cancer and to prevent cancer [54,55]. The use of the mouse model created in this study could enable estimation of the effects of these drugs, thereby indicating appropriate target and drug combinations.

There were some limitations to the present study. First, we were not able to evaluate the impact of DSS-administration on CIN and methylation. Second, the combination of probiotics and prebiotics that we used is only one of many possible combinations. Many studies have investigated strains that are beneficial for intestinal inflammation and immunity, and comparison of a variety of combinations is an important consideration for future research [28–37]. Third, although we used normal colon mucosa to analyze the expression of gene transcripts related to inflammation and tumorigenesis, stromal cells were present among the mucosal epithelial cells because the tissue was collected macroscopically. Therefore, we were unable to obtain a completely uniform evaluation due to cell heterogeneity. Also, this study selected probiotics and prebiotics that have been shown to be useful. The combination of either the *Lactobacillus casei* strain Shirota or *Bifidobacterium breve* strain Yakult as probiotics and oligosaccharide as probiotics may be useful for suppressing enteritis and tumor development. However, the purpose of this study was not to detect the best combination of probiotics and prebiotics, and this will be left for future research.

In conclusion, using *CPC;Apc* mice, we created an inflammation-related colon cancer mouse model in which tumor development is promoted via colitis induced by the administration of DSS. The strength of this model is that it is based on CIN with the single knockout of *Apc*, and does not require the use of carcinogens. Moreover, it is physiologically similar to human carcinogenesis in colorectal cancer and enables observation of the effects of drug administration. Furthermore, the present study demonstrates that synbiotics-treatment suppressed colitis and tumor initiation in this model. The notion in the current study that synbiotics have downregulated IL-6, STAT3, COX-2, and TNF-α genes, which are normally associated with inflammation and tumorigenesis in colon epithelium could possibly disclose new promising therapeutic avenue for patients with colitis-associated colorectal cancer.

## Supporting information

S1 Table Disease activity score assessment (maximum score 12). (DOCX)

S2 Table Histopathological scoring (maximum score 11). (DOCX)

S1 File NC3Rs ARRIVE guidelines checklist. (PDF)
