# Supplementary material for: Synbiotics suppress colitis-induced tumorigenesis in a colon-specific cancer mouse model
Source: PLoS One. 2019 Jun 26;14(6):e0216393. doi: 10.1371/journal.pone.0216393 (PMC6594584; doi:10.1371/journal.pone.0216393)
Supplement: S1 Table — (DOCX) [file pone.0216393.s001.docx]

| **S1 Table. Disease activity score assessment (maximum score 12)** | | | | | | | |
| --- | --- | --- | --- | --- | --- | --- | --- |
|  | |  | |  | |  | |
| **Score** | | **body weight change** | | **stool character** | | **occult blood** | |
| 0 | | no change | | normal | | no blood | |
| 1 | | 1 - 5% weight loss | | soft with well-formed pellets | |  | |
| 2 | | 5 -10% weight loss | | soft without pellets | | partial gross bleeding | |
| 3 | | 10 -20% weight loss | |  | |  | |
| 4 | | >20% weight loss | | diarrhea | | gross bleeding | |
|  | |  | |  | |  | |
