# Supplementary material for: Synbiotics suppress colitis-induced tumorigenesis in a colon-specific cancer mouse model
Source: PLoS One. 2019 Jun 26;14(6):e0216393. doi: 10.1371/journal.pone.0216393 (PMC6594584; doi:10.1371/journal.pone.0216393)
Supplement: S2 Table — (DOCX) [file pone.0216393.s002.docx]

| **S2 Table. Histopathological scoring (maximum score 11)** | | |  |
| --- | --- | --- | --- |
|  |  |  |  |
| **Score** | **inflammation** | **crypt damage** | **ulceration** |
| **0** | rare inflammation cells in the lamina propria | intact crypts | absence of ulceration |
| **1** | increase numbers of granulocytes in the lamina propria | loss of the basal one-third | 1 or 2 foci of ulceration |
| **2** | confluence of inflammatory cells extending into the submucosa | loss of the two-third | 3 or 4 foci ulceration |
| **3** | transmural extension of the inflammatory infiltrate | entire crypt loss | confluent or extensive ulceration |
| **4** |  | change of epithelial surface with erosion |  |
| **5** |  | confluent erosion |  |
